# CCL3 and CCL20-recruited dendritic cells modified by melanoma antigen gene-1 induce anti-tumor immunity against gastric cancer ex vivo and in vivo

**DOI:** 10.1186/1756-9966-29-37

**Published:** 2010-04-27

**Authors:** Songbing He, Liang Wang, Yugang Wu, Dechun Li, Yanyun Zhang

**Affiliations:** 1 Department of General Surgery, the First Affiliated Hospital of Soochow University, 215006 Suzhou, Jiangsu Province, China; 2 Department of General Surgery, the Third Affiliated Hospital of Soochow University, 213000 Changzhou, Jiangsu Province, China; 3 Institute of Health Science and Shanghai Institute of Immunology, Shanghai Institute for Biological Science, Chinese Academy of Science and Shanghai Jiao Tong University School of Medicine, 200025 Shanghai, China

## Abstract

**Background:**

To investigate whether dendritic cell (DC) precursors, recruited by injection of **chemokine ligand 3 (CCL3) and CCL20**, induce anti-tumor immunity against gastric cancer induced by a DC vaccine expressing melanoma antigen gene-1 (MAGE-1) ex vivo and in vivo.

**Methods:**

B6 mice were injected with CCL3 and CCL20 via the tail vein. Freshly isolated F4/80^-^B220^-^CD11c^+ ^cells cultured with cytokines were analyzed by phenotype analysis and mixed lymphocyte reaction (MLR). For adenoviral (Ad)-mediated gene transduction, cultured F4/80^-^B220^-^CD11c^+ ^cells were incubated with Ad-MAGE-1. Vaccination of stimulated DC induced T lymphocytes. The killing effect of these T cells against gastric carcinoma cells was assayed by MTT. INF-γ production was determined with an INF-γ ELISA kit. In the solid tumor and metastases model, DC-based vaccines were used for immunization after challenge with MFC cells. **Tumor size, survival of mice, and number of pulmonary metastatic foci were used to assess the therapeutic effect of DC vaccines**.

**Results:**

F4/80^-^B220^-^CD11c^+ ^cell numbers increased after **CCL3 and CCL20 **injection. Freshly isolated F4/80^-^B220^-^CD11c^+ ^cells cultured with cytokines were phenotyically identical to typical DC and gained the capacity to stimulate allogeneic T cells. These DCs were transduced with Ad-MAGE-1, which were prepared for DC vaccines expressing tumor antigen. T lymphocytes stimulated by DCs transduced with Ad-MAGE-1 exhibited specific killing effects on gastric carcinoma cells and produced high levels of INF-γ ex vivo. In vivo, tumor sizes of the experimental group were much smaller than both the positive control group and the negative control groups (*P *< 0.05). Kaplan-Meier survival curves showed that survival of the experimental group mice was significantly longer than the control groups (*P *< 0.05). In addition, MAGE-1-transduced DCs were also a therapeutic benefit on an established metastatic tumor, resulting in a tremendous decrease in the number of pulmonary metastatic foci.

**Conclusions:**

**CCL3 and CCL20**-recruited DCs modified by adenovirus-trasnsduced, tumor-associated antigen, MAGE-1, can stimulate anti-tumor immunity specific to gastric cancer ex vivo and in vivo. This system may prove to be an efficient strategy for anti-tumor immunotherapy.

## Background

Gastric cancer is one of the most formidable cancers [1]. Although therapies have improved over the years, it is still difficult to treat advanced gastric cancer that has metastasized and spread to the lymph glands. Currently, radical surgery is the only treatment with a curative potential for this disease, and adjuvant chemotherapy or radiotherapy have been widely applied. Nonetheless, control of gastric cancer at an advanced stage still remains difficult [2,3]. Accordingly, new treatment modalities are worth investment to improve 5-year survival rates of patients. One promising approach is immunotherapy.

Dendritic cells (DCs) are professional antigen presenting cells (APC) with the unique capacity to establish a primary immune response against tumor-associated antigens (TAA) [4,5]. This essential role of DCs in cellular immunity has led to development of feasible and effective DC-based vaccines against tumor antigens to eliminate cancer cells. To improve the strategy for DC-based vaccines, it is critical to acquire a large number of appropriate DCs possessing normal function. We have demonstrated that i.v. administration of **chemokine ligand 3 (CCL3) or/and CCL20 **rapidly recruits a group of F4/80^-^B220^-^CD11c^+ ^cells into the peripheral blood. These cells can differentiate into mature DCs [6,7]. We have reported previously that TAA-loaded DCs can stimulate cytotoxic T lymphocytes (CTL) significantly to lyse gastric cancer cells ex vivo [8]. Moreover, DC vaccination induced protective immunity toward the development of gastric cancer in vivo. However, these DC vaccines have not been substantially effective in inducing tumor regression in established gastric cancer. Thus, their therapeutic effects are limited. Despite this, DC-based immunotherapy is considered promising for anti-tumor therapy.

However, new strategies for improved treatment are necessary. Much research has focused upon finding feasible and effective DC-based vaccines. These include pulsing DC with tumor lysates, tumor antigen peptide, or protein; fusing tumor cells with DC; and transducing genes encoding tumor antigen, cytokines, or chemokines into DCs [9]. Melanoma-associated antigen gene-1 (MAGE-1) was initially isolated from the MZ-2 human melanoma cell line [10], which can be recognized by CTL. We and others have previously shown that MAGE-1 is expressed at a high frequency in gastric cancer [11,12], which suggested MAGE-1 may be a target for anti-tumor immunotherapy. In the present study, we demonstrated that F4/80^-^B220^-^CD11c^+ ^DC precursors mobilized by **CCL3 and CCL20 **can induce tumor-specific CTL and elicit potent, therapeutic effects against solid and metastatic tumors when modified with MAGE-1. Together, our results suggest a promising new immunotherapeutic strategy against gastric cancer.

## Methods

### Animals and cell lines

Female BALB/c and C57BL/6 (B6) mice (8-10 weeks) were purchased from the Shanghai Experimental Animal Center, Chinese Academy of Sciences (Shanghai, China). All mice were housed in pathogen-free conditions in the animal center of The Medical College of Shanghai Jiao Tong University (Shanghai, China). Animal care and use were in compliance with institutional guidelines. Mouse forestomach carcinoma (MFC), a mouse gastric cancer cell line, and B16F10, a melanoma cell line of B6 (H-2^b^) mouse origin were purchased from the Shanghai Cell Biology Institutes, Chinese Academy of Sciences (Shanghai, China). Cells were cultured in RPMI (Roswell Park Memorial Institute) medium 1640 (GIBCO, USA) containing 12.5% fetal calf serum (FCS), penicillin G (100 U/ml), and streptomycin (100 μg/ml) at 37°C in a humidified incubator with a 5% CO_2 _atmosphere.

### Major reagents

Human recombinant **CCL3 and CCL20 **expressed in Brevibacillus choshinensis and purified to homogeneity was provided by Dr. Shiro Kanegasaki (Effector Cell Institute, Tokyo, Japan). Murine granulocyte-macrophage colony-stimulating factor (GM-CSF), tumor necrosis factor-α (TNFα), interleukin 4 (IL-4), IL-2, and IL-7 were purchased from Becton Dickinson (New Jersey, USA). Biotinylated anti-F4/80 mAb, Cy-chrome-conjugated streptavidin, phycoerythrin (PE)-labeled anti-B220 mAb, fluorescein isothiocyanate (FITC)-labeled anti-CD11c mAb, rat anti-DEC-205 mAb, FITC-labeled goat anti-rat IgG (Fab)^2 ^antibodies, FITC-labeled mAb against CD40, F4/80, CD11b, or CD80, and PE-labeled mAb against Ia, CD8α, or CD86 were provided by Pharmigen (CA, USA). Mitomycin C (MMC) was purchased from Jingmei Biothe (Shenzhen, China).

### Cell preparation

B6 mice were injected via the tail vein with 1 mg **CCL3 and CCL20 **in 100 μl phosphate-buffered saline (PBS) or with the same dose PBS (control). Peripheral blood (0.8 ml per mouse) was obtained by cardiac puncture from anesthetized mice at the indicated time intervals (0 h, 8 h, 16 h, 24 h, 48 h, 72 h, 120 h) after **CCL3 and CCL20 **injection. Peripheral blood mononuclear cells (PBMCs) were prepared from peripheral blood by density separation with Ficoll. PBMCs were stained with biotinylated anti-F4/80 mAb followed with Cy-chrome-conjugated streptavidin, PE-labeled anti-B220 mAb, and FITC-labeled anti-CD11c mAb for fluorescence-activated cell sorter (FACScan, Becton Dickinson) analysis and sorting of F4/80^-^B220^-^CD11c^+ ^cells. Reanalysis by FACS showed that the purity of these sorted F4/80^-^B220^-^CD11c^+ ^cells was greater than 98%.

### DC development

DCs were generated as described previously [6,13]. Briefly, purified peripheral blood-derived F4/80^-^B220^-^CD11c^+ ^cells from mice injected with **CCL3 and CCL20 **were cultured at a concentration of 3 × 10^5 ^cells/ml in RPMI 1640 medium containing 10% FCS, GM-CSF (4 ng/ml), and IL-4 (10 ng/ml) for 5 d to induce their differentiation into immature DCs. These were cultured further in GM-CSF and TNFα (5 ng/ml) for 3 to 4 d to induce their maturation. Primary DCs were obtained from mouse bone marrow precursors according to a previously established protocol [13]. Mature DCs were observed by light microscopy (Nikon, Japan).

### Immunofluorescence Staining

Before and after culture with GM-CSF and IL-4 for 5 d, and subsequent stimulation with GM-CSF and TNFα for an additional 3 to 4 d, F4/80^-^B220^-^CD11c cells (2 × 10^5 ^to 4 × 10^5 ^cells) were incubated with rat anti-DEC-205 mAb followed by FITC-labeled goat anti-rat IgG (Fab')^2 ^antibodies or directly with FITC-labeled mAb against CD40, F4/80, CD11b, or CD80 and PE-labeled mAb against Ia, CD8α, or CD86 followed by FACS analysis. The instrument compensation was set in each experiment using two-color stained samples.

### Mixed Leukocyte Reaction Assay

MLR was performed in accordance with previous methods [8,14]. Immature and mature DCs were treated with mitomycin C (MMC; 15 μg/ml) in six-well plates at 37°C for 3 h to arrest their proliferation. After several washes with PBS, these stimulator cells were suspended in RPMI 1640 medium containing 10% FCS at concentrations ranging from 1 × 10^2 ^to 5 × 10^4 ^cells/ml. One hundred microliters of the above stimulator cell suspension were added to each well of 96-well plates that contained allogeneic CD4^+ ^T cells (3 × 10^5 ^cells/100 μl per well) that had been magnetically isolated from B6 mice using CD4 Microbeads. Five days later, T-cell proliferation was determined by the MTT method. Fifteen microliters of MTT (5 μg/ml in PBS) was added to each well and the plates were incubated at 37°C for an additional 4 h. The resultant absorbance at 550 nm was read with a microplate immunoreader.

### Recombinant adenoviral vectors and transduction of DC

Recombinant adenovirus (Ad) encoding MAGE-1 (Ad-MAGE-1) was donated by Dr. Yanyun Zhang (Health Science Center of Shanghai Institute for Biological Science, Chinese Academy of Science, China). Ad-MAGE-1 and Ad encoding β-galactosidase (Ad-LacZ) were propagated in 293 cells, purified on a CsCl density gradient, and their titers determined by plaque assay on 293 cells. Aliquots of the adenovirus solutions were stored at - 80°C for use in the following experiments. For Ad-mediated genetic modification, **CCL3 and CCL20**-recruited DCs were incubated with Ad-MAGE-1 or Ad-lacZ at a multiplicity of infection (MOI) of 100 for 2 h at 37°C and then washed twice with complete medium. The above DC vaccines are referred to as DC-Ad-MAGE-1 and DC-Ad-lacZ, respectively. **CCL3 and CCL20**-recruited DCs pulsed with freeze-thawed tumor lysates was performed in accordance with previous methods [8]. The vaccine is referred to as DC-MFC Ag.

### Tumor model and DC-based vaccination

In an established tumor model, 5 × 10^5 ^MFC cells were injected subcutaneously (s.c.) into B6 mice, and the mice were subsequently injected s.c. with DC-Ad-MAGE-1 (1 × 10^6^) on days 5 and 12. As controls, tumor-beating mice were injected with DC-Ad-LacZ, DC-MFC Ag, and untreated DC. Tumor size was evaluated every 2 to 3 d. Survival differences among groups receiving different vaccinations were monitored following challenge with tumor cells. Tumor volume was estimated using the following formula: (short diameter)^2 ^× long diameter × 0.52 [15]. In the pulmonary metastasis model, 5 × 10^5 ^viable MFC tumor cells were injected into B6 mice via tail vein. Mice with pulmonary metastasis were innoculated into the tail vein (i.v.) with 1 × 10^6 ^DC-Ad-MAGE-1 in triplicate at days 3, 7 and 11 after tumor cell injection, respectively. Tumor metastases were evaluated by counting the number of metastases in the lungs of killed mice in macrography.

### CTL assay and interferon gamma (IFN-γ) secretion

Splenic CD3^+ ^T cells (1 × 10^6 ^cells/ml) were cultured in RPMI 1640 containing 10% FCS, then primed ex vivo in the presence of cytokines including IL-2 and IL-7 (5 ng/ml, each) at days 0, 7, and 14 with DC-Ad-MAGE-1 at a stimulator-to-responder cell ratio of 1:20. At day 21 the primed T cells as effector cells were added into 96 well plates containing target MFC or B16F10 tumor cells by serial target cell dilutions (E-T mix, E: T 1:1, 5:1, 10:1, 25:1, 50:1, 100:1). After 20 h, supernatant from each well was collected for measuring cytolytic activity against target cells with a Cytotoxicity Detection Kit (Boehringer Mannheim, Mannheim, Germany). In some experiments, CD3^+ ^T cells were isolated from tumor-free mice that survived for 60 d after tumor cell challenge. These T cells (1 × 10^6 ^cells/ml) were restimulated ex vivo with 1 × 10^5^MMC-treated MFC tumor cells, which were collected for measuring CTL activity and IFN- γ secretion five days later.

### Statistical analysis

Differences were evaluated using Statistical Package for Social Science 11.5 (SPSS 11.5). Survival differences among groups of mice were evaluated with a long-rank test of the Kaplan-Meier survival curves. Statistical tests were two-sided. *P *values < 0.05 were considered to be statistically significant.

## Results

### Identification of CCL3 and CCL20-recruited DC

**Figure 1:**
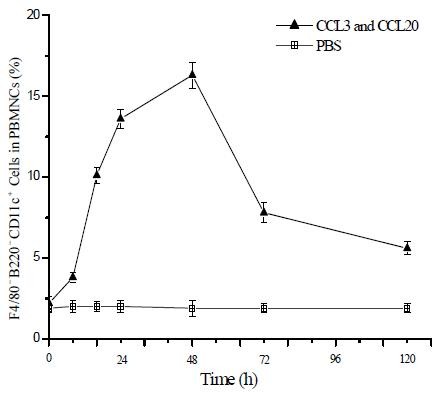
**CCL3 and CCL20 injection recruites F4/80^-^B220^-^CD11c^+ ^cells into the peripheral blood in mice**. B6 mice were injected via the tail vein with 1 mg of CCL3 and CCL20 or with PBS (control). Peripheral blood was obtained by cardiac puncture at the different time intervals (0 h, 8 h, 16 h, 24 h, 48 h, 72 h, 120 h). F4/80^-^B220^-^CD11c^+ ^cells were sorted from PBMNCs and analyzed by FACS. Results are given as means ± SD with 10 mice per group from three independent experiments.

The amounts of F4/80^-^B220^-^CD11c^+ ^cells recruited into the peripheral blood were investigated at different time intervals following **CCL3 and CCL20 **injection. The results showed that numbers of F4/80^-^B220^-^CD11c^+ ^cells gradually increased while there was no change in PBS-injected mice. The percentage of F4/80^-^B220^-^CD11c^+ ^cells reached their highest level (16.55 ± 1.32% of PBMCs) approximately 48 h after **CCL3 and CCL20 **injection (Fig. 1).

**Figure 2:**
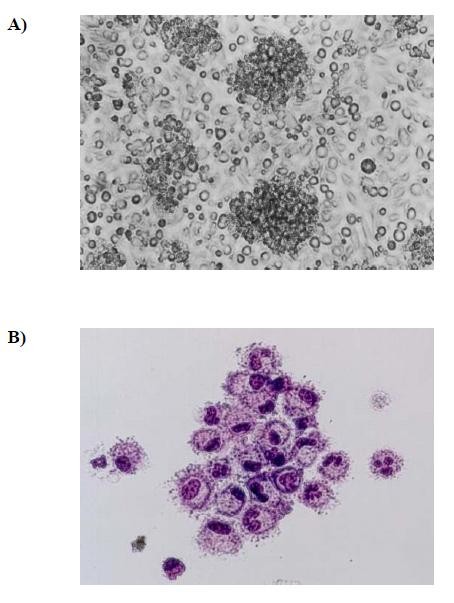
**Morphological characteristics of CCL3 and CCL20-recruited F4/80^-^B220^-^CD11c^+ ^cells before and after culture**. (A), Fresh CCL3 and CCL20-recruited F4/80^-^B220^-^CD11c^+ ^cells were sorted from PBMNCs of mice by FACS and observed by light microscopy (original magnification ×200). (B), These cells were cultured with GM-CSF and TNFα for 5~6 days, then were observed by light microscopy (Giemsa staining was performed, original magnification ×400).

**Figure 3:**
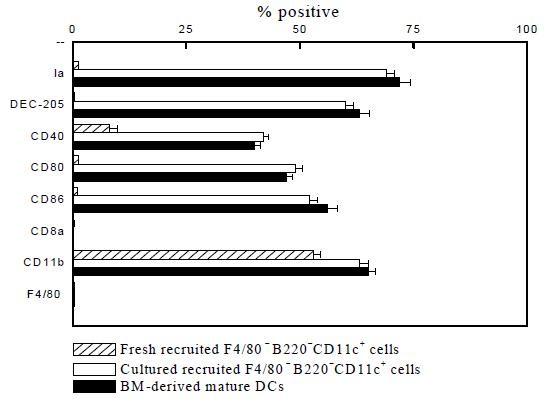
**Immunophenotypic analysis of CCL3 and CCL20-recruited F4/80^-^B220^-^CD11c^+ ^cells**. CCL3 and CCL20-recruited F4/80^-^B220^-^CD11c^+ ^cells cultured for 5~8 days were incubated with PE or FITC-labeled MAbs. The phenotype of these cells was analyzed by immunofluorescence staining as described in the Materials and Methods. Results are given as means ± SD from three independent experiments.

**Figure 4:**
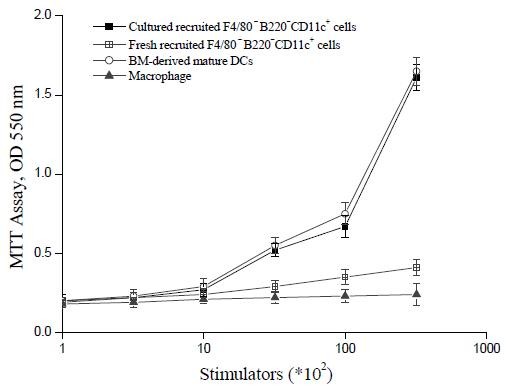
**The capacity of CCL3 and CCL20-recruited F4/80^- ^B220^-^CD11c^+ ^cells to enhance allogeneic MLR**. Allogeneic MLR were performed using splenic T cells purified from B6 mice as responder cells. Fresh and cultured F4/80^-^B220^-^CD11c^+ ^cells were treated with MMC to arrest cell proliferation and were used as stimulator cells at the indicated cell numbers, respectively. Macrophage were used as controls. T cell proliferation was determined with MTT after 5 days of culture. Results are expressed as the mean ± SD of triplicate cultures. All data are representative of three independent experiments.

The **CCL3 and CCL20**-recruited F4/80^-^B220^-^CD11c^+ ^cells were next examined by morphology, phenotype analysis, and MLR. Cells were compared morphologically before and after culturing with GM-CSF, IL-4, and TNFα. Light microscopy showed that culturing with cytokines resulted in large cells with oval or irregularly shaped nuclei and many small dendrites (Fig. 2, **compare panel B to panel A)**. Phenotypically, FACS analysis showed that fresh (i.e., uncultured) F4/80^-^B220^-^CD11c^+ ^cells expressed moderate levels of CD40; low levels of Ia, CD80, CD86, and DEC-205 molecules; and were negative for F4/80 and CD8α antigen (Fig. 3). Functionally, these cells were unable to stimulate allogeneic T cells in a MLR assay (Fig. 4). By contrast, cultured F4/80^-^B220^-^CD11c^+ ^cells expressed high levels of Ia, CD86, CD80, and DEC-205 antigen (Fig. 3) and acquired the capacity to enhance allogeneic T cell proliferation as effectively as mature, BM-derived DCs (Fig. 4).

### Generation of tumor-specific CTL induced byDC-Ad-MAGE-1 ex vivo

**Figure 5:**
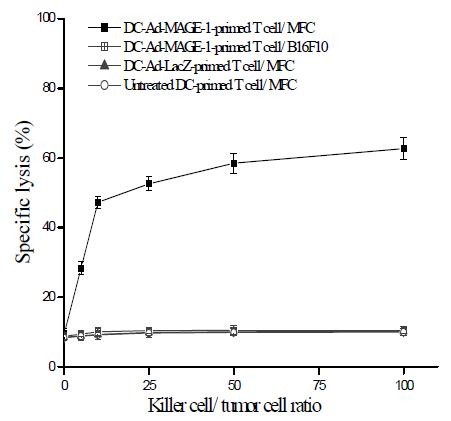
**Generation of tumor-specific CTLs ex vivo**. Splenic CD3^+ ^T cells were isolated from B6 mice with MACS. T cells were primed with MAGE-1-modified DCs as described in Materials and Methods. DC-Ad-LacZ and untreated DCs were used as controls. Primed T cells (effector cells) were titrated by serial dilution, then mixed with MFC or B16F10 target cells, and their lytic activity was assayed. Results are given as means ± SD from three independent experiments.

To study the potential of **CCL3 and CCL20**-recruited DCs in anti-tumor immunity ex vivo, DC-MAGE-1 were employed after five days of culture with GM-CSF and IL-4. Splenic T cells from naïve mice were primed ex vivo with DC-Ad-MAGE-1 in the presence of IL-2 and IL-7 to elicit cytolytic reactivity against tumor cells. When T cells primed with DC-Ad-MAGE-1 were added to tumor cells, they were able to efficiently and specifically lyse MFC, but not B16F10 tumor cells, which do not express MAGE-1. The results also showed that T cells primed with DC-Ad-LacZ or untreated DC did not induce specific CTL (Fig. 5). The results of CTL responses ex vivo suggested that vaccination with MAGE-1-modified DC may prove useful for anti-tumor immunotherapy in vivo.

### A therapeutic effect mediated by DC-Ad-MAGE-1 in vivo

**Figure 6:**
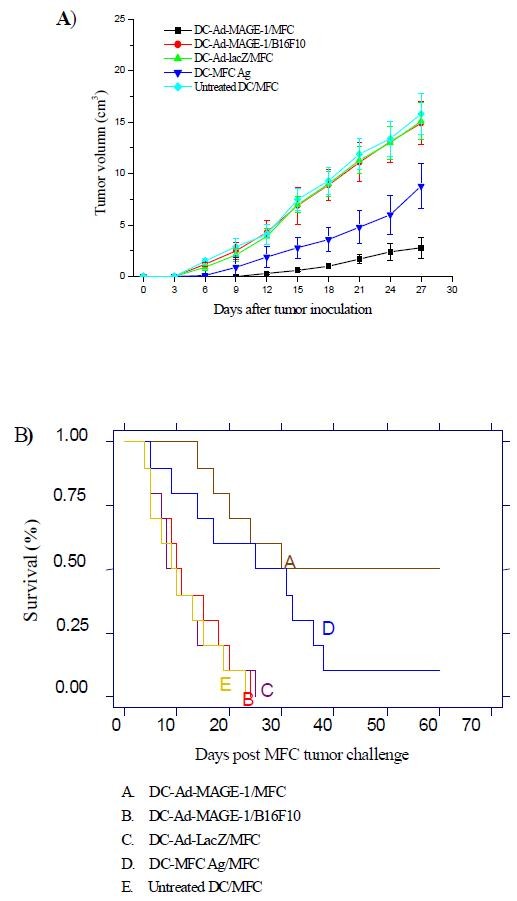
**Inhibition of tumor growth in tumor-bearing mice by immunization with MAGE-1-modified, CCL3 and CCL20-recruited DC vaccine**. (A), Each of 10 mice in a group was challenged s.c. with 1 × 10^5 ^viable MFC tumor cells. Mice were subsequently injected s.c. with DC-Ad-MAGE-1 5 days later. As controls, tumor-bearing mice were injected with DC-Ad-LacZ, DC-MFC Ag, or untreated DCs. Survival was observed over time after immunization of mice harboring preexisting tumors. Survival rate was compared with a long-rank test of Kaplan-Meier curves. (B), Tumor growth was measured every 2~3 days after the second immunization. Data are given as means ± SD of 10 mice per group from three independent experiments.

Therapeutic potential of DC-Ad-MAGE-1 was further explored with an established tumor model. 5 × 10^5 ^MFC or B16F10 tumor cells were implanted s.c in B6 mice, and tumor-bearing mice were injected with different modified or unmodified DCs on days 5 and 12. Fig. 6A shows that tumor growth was significantly inhibited in mice vaccinated with DC-Ad-MAGE-1. For example, tumor volumes on day 27 were as follows: untreated DC control 14.98 ± 1.81 cm^3^, DC-Ad-LacZ control 15.44 ± 1.99 cm^3^, DC-MFC Ag control 7.79 ± 1.55 cm^3^, DC-Ad-MAGE-1 3.46 ± 1.12 cm^3^, DC-Ad-MAGE-1 vs. the other control groups (*P *< 0.05). Half of the tumor-bearing mice immunized with DC-Ad-MAGE-1 survived in a period of over 60 days. By contrast, only 10% of the tumor-bearing mice immunized with DC-MFC Ag survived; all mice from the other control groups succumbed to growing tumors within 25 days, thus providing no therapeutic effect (Fig. 6B). The differences between the DC-Ad-MAGE-1 group and all control groups were statistically significant (*P *< 0.05).

**Figure 7:**
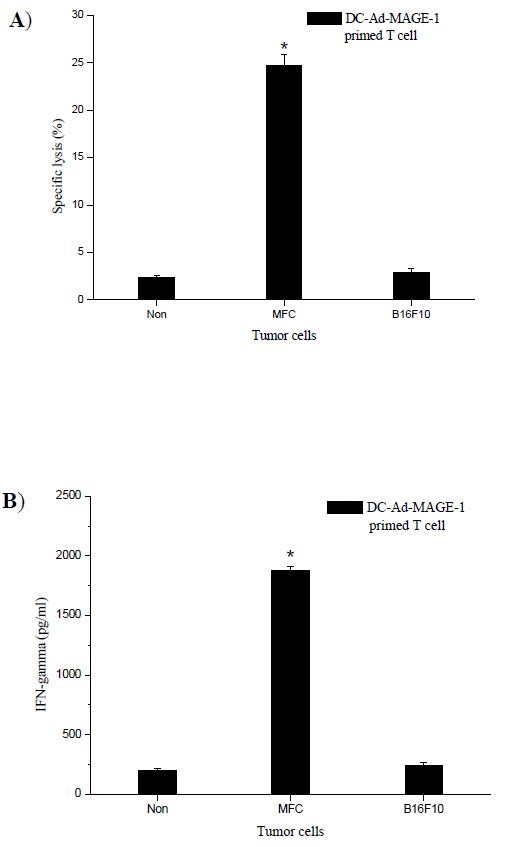
**Assay for tumor-specific, CTL activity and IFN-γ secretion in immunized mice**. (A), Splenic T cells from immunized mice were restimulated ex vivo by culturing with MMC-treated, MFC tumor cells. The restimulated T cells (effector cells) were incubated with target MFC or B16F10 cells for 20 h. Cytolytic activity (lysis) was determined. (B), Supernatants were collected for IFN-γ assay. All data are shown as means ± SD for 10 mice per group and are representative of three independent experiments. * *P *< 0.05.

To confirm that tumor-specific CTLs had indeed been generated in the immunized mice, the following evaluation was performed. Spleen T cells from mice immunized s.c with DC-Ad-MAGE-1, and thus rendered tumor-free after MFC tumor challenge, were restimulated ex vivo with irradiated tumor cells and tested for cytolytic activity. As shown in Fig. 7A, these effector cells efficiently lysed MFC, but not B16F10 tumor cells. Control spleen T cells from naive mice stimulated with irradiated MFC tumor cells failed to demonstrate CTL activity. Furthermore, splenic CD3^+ ^T cells derived from those mice that survived MFC challenge produced high levels of IFN-γ, but not when stimulated with B16F10 cells (Fig. 7B). This observation indicates that the vaccination effect of DCs expressing the tumor antigen gene is antigen specific.

Finally, administration of DC-Ad-MAGE-1 was tested as a possible therapeutic benefit for distant, established visceral metastases. In this treatment model, the benefit of **CCL3 and CCL20-**recruited DCs as a tumor treatment was quantified by counting metastatic foci in pulmonary tumor-bearing mice. These were established by i.v. administration of 5 × 10^5 ^viable MFC tumor cells. Metastatic lung tumors were observed at day 3 after tumor cell implantation. Subsequently, tumor-bearing mice were treated with 1 × 10^6 ^DC-Ad-MAGE-1 cells in triplicate at days 3, 7 and 11 after injection of tumor cells. As controls, mice were treated to the same regimen with either DC-Ad-LacZ, DC-MFC Ag, or untreated DCs. Visible lung metastases in these mice were counted in macrography at day 21 after tumor cell inoculation. Mice treated with DC-Ad-MAGE-1 showed a dramatic reduction in the number of lung metastatic foci. However, a decrease did not appear in mice receiving the control treatments (Table 1).

**Table 1:** Treatment of distant metastatic tumors with MAGE-1-modified DC vaccines

Treatment	Number of Lung metastases
DC-Ad-MAGE-1	*31.38 ± 2.26
DC-Ad-LacZ	120.75 ± 2.71
DC-MFC Ag	77.25 ± 3.37
Untreated DC	124.38 ± 3.58

* P < 0.05, DC-Ad-MAGE-1 versus the other control groups.

## Discussion

We have demonstrated that after injection of **CCL3 and CCL20**, F4/80^-^B220^-^CD11c^+ ^DC precursors are quickly recruited into the peripheral blood. Furthermore, these **CCL3 and CCL20**-recruited DCs, when modified with tumor antigen gene MAGE-1, could induce not only an effective CTL response against gastric cancer cells ex vivo but also therapeutic, anti-tumor immunity in both subcutaneous tumor and pulmonary metastatic tumor models.

Among many different immunotherapeutic strategies currently being evaluated, DC-based vaccination has attracted particular attention as a proven safe and potent therapy against tumors [14,16]. Induction of tumor immunity can be initiated by effectors of innate immunity and can be further developed by cells of adaptive immunity, with DCs playing a central regulatory role. Several steps are involved including (a) recognition of tumor molecules by DC precursors, (b) direct and IFN-γ-mediated killing of transformed cells by NK/NK T cells activated by DCs, (c) capture and cross presentation of released TAA by immature DCs, (d) selection and activation of TAA-specific T cells, as well as nonspecific effectors including macrophages and eosinophils, and (e) homing of TAA-specific T cells to the tumor site and recognition leading to elimination of tumor cells [16]. DC-based vaccination had presented efficient anti-tumor activity in numerous tumor models and in clinical studies. Kono K [17] reported that vaccines using DCs pulsed with HER-2/neu-peptides may represent a novel treatment of gastric cancer patients.

DC migration in vivo involves three steps: mobilization into the blood, recruitment from blood to peripheral tissues, and remobilization from peripheral to lymphoid tissues. Once there, immature DCs finally differentiate into fully mature DCs to promote immune responses. Although the first step has not received much attention, it is important to understand how this step is regulated in order to understand the pathologic role of DCs in various inflammatory diseases and in tumor development. Chemokines selectively direct the trafficking of subsets of leukocytes into various tissues in homeostasis as well as inflammatory states in vivo [18]. The capacity of DCs to migrate to sites of inflammation, where they capture antigens and subsequently migrate to local lymph nodes, is regulated by the expression of different chemokines and chemokine receptors [19,20].

Mobilization of DCs and DC precursors into peripheral blood is of particular interest in research related to DC-based immunotherapy. We have demonstrated that murine F4/80^-^B220^-^CD11c^+ ^DC precursors rapidly appear in peripheral blood when animals are injected i.v. with CCL3 and CCL20 [7]. These F4/80^-^B220^-^CD11c^+ ^cells subsequently differentiate into mature DCs when cultured ex vivo with GM-CSF and TNFα. The resultant DCs present the typical morphological characteristics, phenotypes, and antigen-presenting functions of DCs (as assessed in MLR assays). **Because injections of CCL3 and CCL20 did not induce any detectable inflammatory response or liver injury in vivo (data not shown), we believe it is possible that CCL3 and CCL20 could be employed to efficiently recruit DC precursors for the purpose of DC-based cancer therapy**.

There are two considerably important factors involved in DC-based vaccination in the clinic: one is the way to effectively and practically obtain abundant DCs in peripheral blood; the other is a method to effectively modify DCs used as vaccines for tumor rejection and therapy [21]. Successful genetic modification of murine **CCL3 and CCL20**-recruited DCs with adenoviral vectors was demonstrated. Adenovrial-based gene therapy has many advantages over other forms of TAA delivery [22]. Adenoviral vectors allow local, highly efficient, albeit transient, gene expression, generating high-level, but limited, cytokine production in treated tumors. Adenoviral vectors are transduction agents in a heterogeneously growing population of tumor cells. In this study, murine DCs were transduced using cocultivation with adenoviral vectors. The murine **CCL3 and CCL20 **-recruited DCs were transduced with MAGE-1 at different MOI and different time intervals in culture. DCs transduced with MAGE-1 at an MOI of 100 showed limited toxicity and maximal production of MAGE-1 (data not shown).

In this study, CCL3 and CCL20-recruited DCs were modified with a tumor antigen gene and used as vaccines for an anti-tumor immune response ex vivo and in vivo. Ex vivo, when T cells were primed with MAGE-1-modified DCs and added to tumor cells, they were able to lyse tumor cells efficiently and specifically. High cytolytic activity in association with a Th1-type response could possibly contribute to the profound anti-tumor effects that we observed. In vivo, vaccination with CCL3 and CCL20-recruited DCs modified with MAGE-1 remarkably inhibited subcutaneous tumor growth and size. This observation suggests the treatment potential of these cells as vaccines. In addition, splenic T cells obtained from mice vaccinated with DC-Ad-MAGE-1 produced high levels of IFN-γ and showed specific cytotoxic activity. By contrast, responses induced by nontransduced DCs and TAA-loaded DCs were far less potent. **While most DC-based vaccination strategies target solid, non-metastatic tumors, our vaccination strategy employing TAA gene-modified DCs revealed efficacy against metastatic tumors as well. Future work will address the idea that this approach may be a viable one for treatment of gastric cancers in patients**.

## Conclusion

In this study, we demonstrated that F4/80^-^B220^-^CD11c^+ ^DC precursors were rapidly recruited into the peripheral blood by administration of **CCL3 and CCL20 **in mice. This is essential for preparing DC-based vaccines against tumors. Importantly, vaccination with these DCs modified with MAGE-1, could elicit specific CTL responses to gastric cancer cells, and led to tumor rejection ex vivo and in vivo. These results suggest that an evaluation of this DC-based immunotherapy strategy for gastric cancer patients is an important next step.

## Competing interests

The authors declare that they have no competing interests.

## Authors' contributions

SH carried out literature research, experimental studies and data acquisition, participated in the study design, and drafted the manuscript. LW participated in the design of the study and performed the statistical analyses. YW carried out immunoassays, data acquisition, and manuscript editing. DL and YZ conceived of the study, participated in its design and coordination, and assisted writing the manuscript. All authors read and approved the final manuscript.
